# Plant Growth-Promoting Fungi as Biocontrol Tool against Fusarium Wilt Disease of Tomato Plant

**DOI:** 10.3390/jof8080775

**Published:** 2022-07-26

**Authors:** Mohamed S. Attia, Amer M. Abdelaziz, Abdulaziz A. Al-Askar, Amr A. Arishi, Ahmed M. Abdelhakim, Amr H. Hashem

**Affiliations:** 1 Botany and Microbiology Department, Faculty of Science, Al-Azhar University, Cairo 11884, Egypt; drmohamedsalah92@azhar.edu.eg (M.S.A.); amedhakem901@gmail.com (A.M.A.); 2 Department of Botany and Microbiology, Faculty of Science, King Saud University, Riyadh 12372, Saudi Arabia; 3 School of Molecular Sciences, The University of Western Australia, Perth, WA 6009, Australia; 22650755@student.uwa.edu.au

**Keywords:** antifungal activity, *Fusarium oxysporum*, plant growth-promoting fungi, biocontrol, isozymes

## Abstract

Plant growth-promoting fungi (PGPF) improve plant health and resist plant pathogens. The present study was carried out to biocontrol tomato Fusarium wilt using PGPF through antifungal activity and enhance tomato plant immune response. Four PGPF were identified genetically as *Aspergillus flavus*, *Aspergillus niger*, *Mucor circinelloides and Pencillium oxalicum*. In vitro antagonistic activity assay of PGPF against *Fusarium* *oxysporum* was evaluated, where it exhibited promising antifungal activity where MIC was in the range 0.25–0.5 mg/mL. Physiological markers of defense in a plant as a response to stimulation of induced systemic resistance (ISR) were recorded. Our results revealed that *A. niger*, *M. circinelloides*, *A. flavus* and *P. oxalicum* strains significantly reduced percentages of disease severity by 16.60% and 20.83% and 37.50% and 45.83 %, respectively. In addition, they exhibited relatively high protection percentages of 86.35%, 76.87%, 56.87% and 59.06 %, respectively. With concern to the control, it is evident that the percentage of disease severity was about 87.50%. Moreover, the application of *M. circinelloides*, *P. oxalicum*, *A. niger* and *A. flavus* successfully recovered the damage to morphological traits, photosynthetic pigments’ total carbohydrate and total soluble protein of infected plants. Moreover, the application of tested PGPF enhanced the growth of healthy and infected tomato plants.

## 1. Introduction

Tomato diseases are common among other plant diseases where the great losing economic value of the tomato crop occurs [1]. Tomato is one of the most important agricultural crops in the world as it is a rich source of vitamins A and C [2,3]. The tomato crop is an important part of the Mediterranean and other traditional diets. However, there is a great concern for humanity in terms of food security, as demographic projections indicate that the population will rise to reach 9.5 billion by 2050. Under the threat of climate change and the spread of pathogens, improving crop productivity and avoiding the use of chemical pesticides is a major issue for the agricultural industry [4]. Fungal diseases are among the most dangerous biological stresses that cause severe damage to agricultural crops in many countries [5]. Plant diseases are one of the most important global problems that threaten the agricultural wealth, as they cause huge losses in agricultural production, in addition to reducing the quality of the product, as well as the secretion of toxins or toxins that cause poisoning and multiple serious diseases that affect humans and animals that eat this affected product [6]. Fungal pathogens are the main destructive and distributed plant pathogen, representing about 80% of crop diseases that cause serious crops and economic inhibition [7]. Soil-borne fungi are difficult to control due to the soil system being a complex system in which different pathways take place in a short time [8]. In light of the climatic changes that the world is exposed to, plants have become more susceptible to disease. Scientific reports have proven that the plant has the ability to defend itself against disease through resistance to prevent or limit the progression of the pathogen, either by synthetic means, that is, due to the appearance or anatomical structure, or by chemical means, i.e., the presence of certain substances that inhibit the pathogen [9] One of the most famous pathogens of fungal diseases, *Fusarium oxysporum*, causes a negative impact on crops, especially vegetables [10,11]. However, fusarium wilt disease, caused by *Fusarium oxysporum*, causes severe injury through all phases of tomato growth [12]. In Egypt, the damages to tomato crop production due to *F. oxysporum* wilt raised up to 67% of the total planted area [13]. The traditional strategies to limit the wilt disease, including the use of antifungals and crop cycle, have not been effective due to fungal conidia staying viable for abundant years and the harmful effects of pesticide residues on human health; thus, it is necessary to improve new efficient control plans that do not affect the environmental safety [14]. In light of the importance of the tomato crop, the development of new management methods to improve resistance to biological stresses such as fungal, bacterial, viral and insect infections may help in enhancing global food production that is safe and free of harmful pesticides. The use of growth-stimulating microorganisms is a common strategy for researchers to enhance and improve the defense capacity and physiological immunity of plants as well as the bioavailability of minerals in the soil [15,16]. Plants can induce disease resistance to a group of pathogenic [17]. It is worth noting that the endophyte is one of the most important biological means for stimulating plant immunity against pathogens [18,19,20]. Growth-promoting microorganisms were used as bio-nutrients to effectively increase nutrition content in crops [21,22] by improving the properties of the soil and secreting enzymes that facilitate the uptake of nutrients by the plant without leading to environmental pollution, and to achieve higher efficiency for enhancement the crops quality and quantity [16,23,24]. Plant growth promoting fungi have many mechanisms for controlling fungal phytopathogens such as antioxidant compounds that recover the oxidative burst, minimizing the mycotoxins production [23]. Biological control is an alternative strategy to control of the *Fusarium* wilts by using Antagonistic nonpathogenic microorganisms, that have potency to minimize or recover the harmful effects in numerous crops [24]. Recent studies forcefully favored the application of biological control as safety approach for human and environment to control *F. oxysporum* in Egypt [25]. Stimulated resistance is the physiological state produced by specific eco-friendly stimuli that have antimicrobial potency against a broad range of plant pathogens, including fungi [26]. Plant growth can be enhanced by non-pathogenic fungi through different mechanisms, such as systemic resistance stimulation and plant nutrition improvement, in addition to their toxicity to plant pathogens [27,28,29]. Many plants’ Rhizosphere soils were used for the isolation of several microbial strains that enhanced plant protection by improving the antimicrobial potency. Herein, this study aimed to use PGPF targeting for plant growth promotion, as well as antifungals, on *F. oxysporum* in vitro and in vivo. Our study provided the possibility of using PGPF as a safe and alternative method to control the tomato *Fusarium* wilt.

## 2. Materials and Methods

### 2.1. Tomato Plant

Well-recognized four-week-old tomato seedlings (*Solanum Lycopersicon* L. cv. Castlerock II PVP) were obtained from the agricultural research center (ARC), ministry of agriculture, Giza, Egypt.

### 2.2. Source and Maintenance of the Pathogen

*F. oxysporum f.* sp. *Lycopersici* RCMB008001 was obtained from the Regional Center for mycology attAl-Azhar University and then confirmed by pathogenicity test according to Hibar et al. [30]_ENREF_44; the pathogenicity test and inoculum were achieved according to Aldinary and Abdelaziz [28].

### 2.3. Isolation and Identification of PGPF

Four fungal strains, F2, F5, F13 and F25, were isolated and identified morphologically in our previous study [31]. In the current study, these PGPF strains were identified genetically using ITS. The genomic DNA was isolated and purified using Quick-DNA Fungal Microprep Kit (Zymo research; D6007), and molecular identification was achieved by internal transcribed spacer (ITS) region according to [Khalil et al. [32]].

### 2.4. Extraction of Fungal Metabolites Using Ethyl Acetate

Four fungal strains, *M. circinelloides*, *A. flavus*, *A. niger* and *P. oxalicum*, were cultured separately in the potato dextrose broth (PDB) at 26 ± 2 °C under static conditions for 14 days. Afterward, the culture was filtered using Whatman No 1 filter paper. The obtained culture filtrate was mixed with ethyl acetate 1:1 ratio and collected in the upper organic layer. Further, the extraction process was repeated 3–4 times, pooled together and condensed at 45 °C using a rotary evaporator. Finally, ethyl acetate extracts were collected and stored at room temperature [33].

### 2.5. In Vitro Antifungal Activity of PGPF

The well diffusion method was applied to study the antifungal activity of ethyl acetate fungal extracts of *M. circinelloides*, *A.* *flavus*, *A. niger* and *P. oxalicum*. *F. oxysporum* was inoculated on PDB and then incubated at 28 ± 2 °C for 3–5 days. Inoculum of *F. oxysporum* was spread thoroughly on the sterilized solidified PDA medium. Wells (7 mm) were filled with 50 µL of each fungal extract (4 mg/mL) and were put in each well. The culture plates were incubated at 25 °C for 7 days, and the zones of inhibition were observed and measured. Moreover, minimum inhibitory concentration (MIC) was carried out, where different concentration of each fungal extract (4, 2, 1, 0.5, 0.25 and 0.125 mg/mL) was put in wells to detect MIC [34].

### 2.6. In Vivo Study

Applied PGPF was applied one week before infection with *F.* *oxysporum*. The pot experiments were achieved at the plant garden of the Botany and Microbiology Department, Faculty of Science, Al-Azhar University. Seedlings were planted in 10 groups (each group contained 6 plants) as follows: (T1) plants without any treatment (healthy control), (T2) plants infected with *F.* *oxysporum* (infected control), (T3) healthy plants treated with *M. circinelloides*, (T4) infected plants treated with *M. circinelloides*, (T5) healthy plants treated with *P. oxalicum*, (T6) infected plants treated with *P. oxalicum*, (T7) healthy plants treated with *A. niger*, (T8) infected plants treated with *A. niger*, (T9) healthy plants treated with *A. flavus* and (T10) infected plants treated with *A. flavus*. The morphological and physiological indicators for resistance were assayed after 45 days of inoculation.

### 2.7. Disease Severity and Percentage the Protection

The disease symptoms were observed 45 days post-inoculation. Disease severity and percentage of the protection of PGPF were estimated by using five classes: (0) no symptoms, (1) slight yellowing of lower leaves, (2) modest yellowish, (3) complete wilted and browning of vascular bands and finally (4) stunted and death of plants. The percent disease index (PDI) was calculated by the formula: PDI = (1n1 + 2n2 + 3n3 + 4n4)·100/4nt, where n1–n4 is the number of plants in each class and nt is the total number of plants. Protection% = A − B/A × 100%, where A = PDI in infected control plants and B = PDI in treated infected plants [25].

### 2.8. Resistance Indicators

Morphological resistance indicators (shoot length, root length and the number of leaves) were recorded. Photosynthetic pigments were tested according to Vernon and Seely [35]. In such a method, 0.5 g of fresh leaves was ground in a mortar using 50 mL of 80% aqueous acetone (*v/v*). The homogenate was filtrated by Whatman No.1 filter paper. Then the filtrate was made up to 100 mL using 80% acetone, and the developed green color was measured spectrophotometrically at 665, 649 and 470 nm. The concentrations of chlorophyll (a), (b) in plant tissue were calculated using the equations mg chlorophyll (a)/g tissue = 11.63(A665) − 2.39(A649), mg chlorophyll (b)/g tissue = 20.11(A649) − 5.18(A665) and Carotenoids = 1000 × O.D_470_ − 1.82 C_a_ − 85.02 C_b_/198 = mg/g fresh weight. “A” denotes the reading of optical density. The method Lowry, O.H., et al. [36] was used to determine the soluble protein content. In this technique, The dried tomato leaves were ground to a fine powder. An amount of 1g of the dried powder was put in a 100 mL capacity conical flask, to which 5 mL of 2% phenol water and 10 mL distilled water were added. The mixture was shaken and kept overnight before being filtered, and the filtrate was made up of 50 mL of distilled water. An amount of 0.1 mL of the extract was mixed with 5 mL of solution (50 mL of solution 2% NaCO_3_ in 0.1 N NaOH mixed with 1 mL of solution 0.5 g CuSO_4_ in 1% sodium potassium tartrate) and left to stand for 10 min. Then, 0.5 mL of folin reagent (BDH) with distilled water in the proportion of 1:3 (*v/v*) was rapidly added and mixed with the contents and left to stand for an additional 30 min. The optical density of the resulted color was then read at the wavelength of 750 nm. The soluble sugar content was estimated according to the method used by [37]. For soluble sugars extraction, the dried shoots (0.5 g) were mixed with 2.5 mL of 2% phenol and 5 mL of 30 % trichloroacetic acid, then filtrated throughout filter paper: 1 mL of the filtered was then mixed with 2 mL of anthrone reagent (2 g anthrone/L of 95% H_2_SO_4_), and the color (blue–green) was measured at 620 nm. Totally dry shoot phenol content was measured using the Diaz, D.H. and G.C. Martin [38] technique. The proline content was assessed in the dry leaves according to Bates et al. [39]. Furthermore, Peroxidase activity (POD) and Polyphenol Oxidase (PPO) were assayed according to Bates et al. and Srivastava [40].

### 2.9. Isozymes Electrophoresis

Innate polyacrylamide gel electrophoresis was achieved to detect isozymes modifications in response to treatments. POD and PPO isozymes were assessed by the methods of Matta, A. and A. Dimond; Barceló et al. [41,42]. An amount of 0.5 g of fresh leave samples homogenized in 1 mL of (10% glycerol) was then centrifuged at 10,000 rpm for 5 min. A volume of 50 μL extract of each sample was mixed with 20 μL sucrose and 10 μL bromophenol blue. Then, a volume of 80 μL from this mixture was applied to each well. The Gel Doc VILBER LOURMAT approach was used to evaluate and investigate gels. While the gels were saturated, the banding shape was videotaped, and the band’s number was declared in each gel lane and computed and correlated with each other. The Helena Densitometer Model Junior 24 was used to determine quantitative band quantity and strength changes.

### 2.10. Statistical Analyses

Valuation of pilot data was accomplished by using a one-way analysis of variance (ANOVA). Additionally, means differences by Duncan’s multiple range test and the (L.S.D) at a 5.0% level of probability were obtained using Co State software(CoHort, Monterey, CA, USA) [43,44].

## 3. Results

### 3.1. Molecular Identification of PGPF

**Figure 1:**
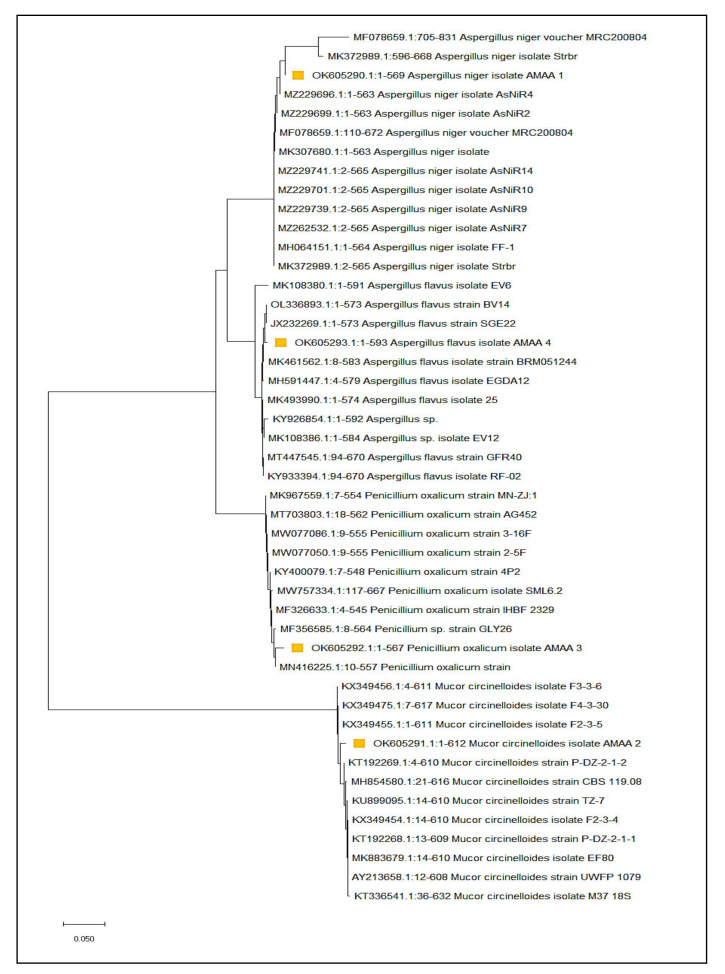
Phylogenetic tree of *A. flavus*, *P. oxalicum*, *M. circinelloides* and *A. niger* with accession numbers OK605293, OK605292, OK605291 and OK605290.

Molecular identification confirmed that F2, F5, F13 and F25 resembled *Aspergillus flavus*, *Penicillium oxalicum*, *Mucor circinelloides* and *Aspergillus niger* with a similarity of 99%, respectively. The sequences of the four strains *A. flavus*, *P. oxalicum*, *M. circinelloides* and *A. Niger* were recorded in the gene bank with accession numbers OK605293, OK605292, OK605291 and OK605290, respectively, as shown in Figure 1.

### 3.2. In Vitro Antifungal Activity of PGPF Strains against F. oxysporum

**Figure 2:**
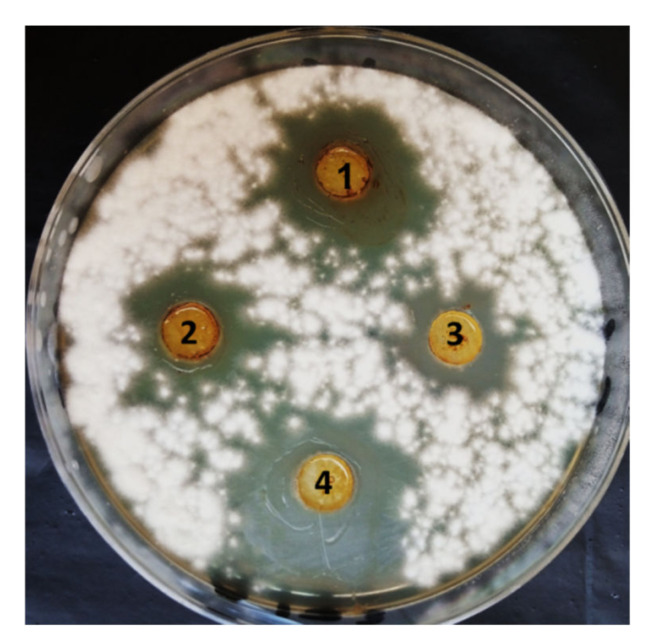
Inhibition zones of ethyl acetate extracts of *P. oxalicum* (**1**), *A. flavus* (**2**), *A. niger* (**3**) and *M. circinelloides* (**4**) against *F. oxysporum* using agar well diffusion method.

**Figure 3:**
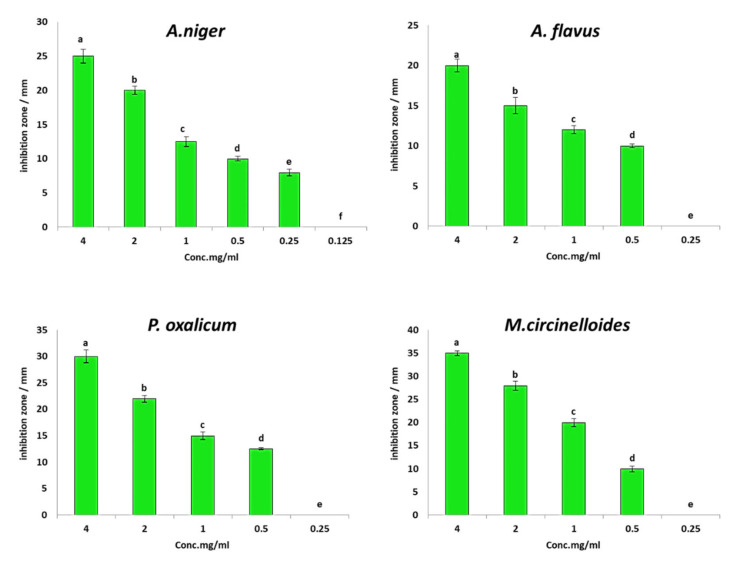
Minimum inhibitory concentrations of the four fungal extracts against *F. oxysporum*.

Antifungal activity of ethyl acetate fungal extracts of *M. circinelloides*, *P. oxalicum A. flavus* and *A. niger* were evaluated against *F. oxysporum* using the agar well diffusion method as shown in Figure 2. The results revealed that the four extracts have promising antifungal activity against *F. oxysporum*, where inhibition zones of fungal extracts of *M. circinelloides*, *P. oxalicum*, *A. flavus* and *A. niger* at concentrations of 4 mg/mL were 31, 22.8, 20.4 and 15.5 mm, respectively (Figure 2). Moreover, different concentrations (0.125–4.0 mg/mL) of the four extracts were evaluated to detect MIC for each extract, as shown in Figure 3. Results illustrated that MIC of *A. niger* was 0.25 mg/mL, while it was 0.5 mg/mL for each extract of *M. circinelloides*, *P. oxalicum* and *A. niger*. The four extracts of *M. circinelloides*, *P. oxalicum*, *A. flavus* and *A. niger* exhibited in vitro antifungal activity; therefore, the MIC concentrations of the four extracts were used for pot experiments.

### 3.3. Estimation of Tomato Systemic Resistance Induced by PGPF against Fusarium Wilt

#### 3.3.1. Disease Severity (DS) and Protection%

The results in the table indicated that *Fusarium oxysporum* has a highly destructive effect on tomato plants by PDI 87.5%. On the other hand, all applied PGPF were reduced PDI (Table 1), where *A. niger*, *M. circinelloides*, *A. flavus* and *P.* *oxalicum* isolates reduced the disease severity percentages by 16.6, 20.83, 37.5 and 45.83%, respectively. In addition, PGPF revealed high protection percentage of 81.3, 76.19, 59.06, 57.14 and 47.62% against *Fusarium* wilt.

#### 3.3.2. Growth Indicators

**Figure 4:**
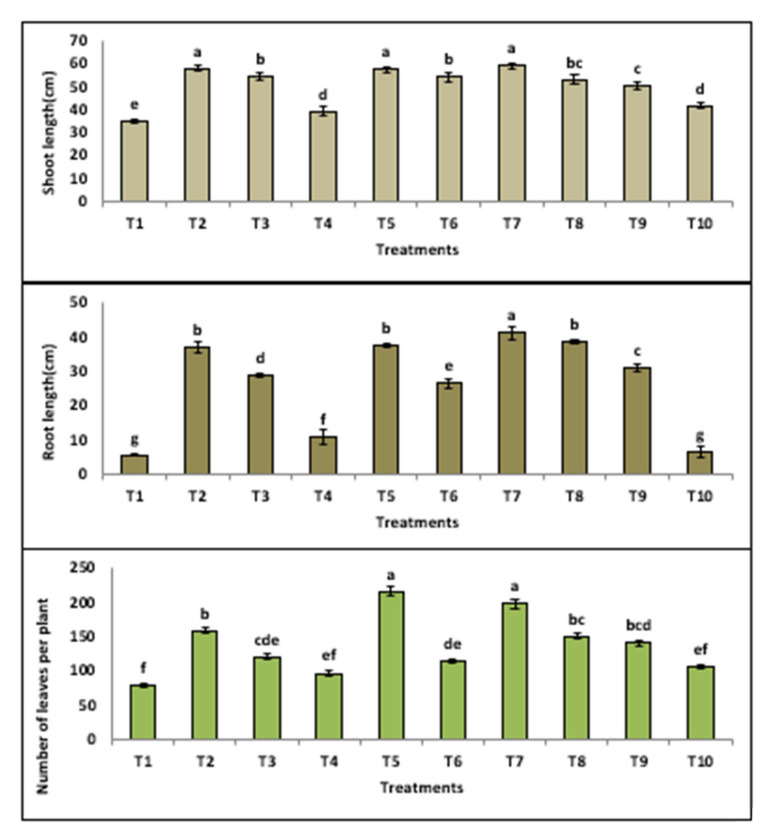
Morphological indicators of tomato plant (shoot length, root length and number of leaves) treated with PGPF. (T1) infected control, (T2) healthy control, (T3) healthy + *M. circinelloides*, (T4) infected + *M. circinelloides*, (T5) healthy + *P. oxalicum*, (T6) infected + *P. oxalicum*, (T7) healthy + *A. niger*, (T8) infected + *A. niger*, (T9) healthy + *A. flavus*, (T10) infected + *A. flavus*.

It is clear from the Figure 4 that infected tomato plants with *F. oxysporum* showed significant loss of shoot length by (40.4%), root length by (85%) and the number of leaves by (50.6%) in comparison to healthy control plants. The results revealed that various growth parameters (shoot length, root length and the number of leaves) were significantly increased by using the extracts of all PGPF, as shown in Figure 4. On the other hand, infected plants treated with tested fungal isolates (*A. niger*, *A.* *flavus*, *M. circinelloides* and *P.* *oxalicum*) showed hopeful recovery, and the best treatment was *P. oxalicum*.

#### 3.3.3. Photosynthetic Pigment

**Figure 5:**
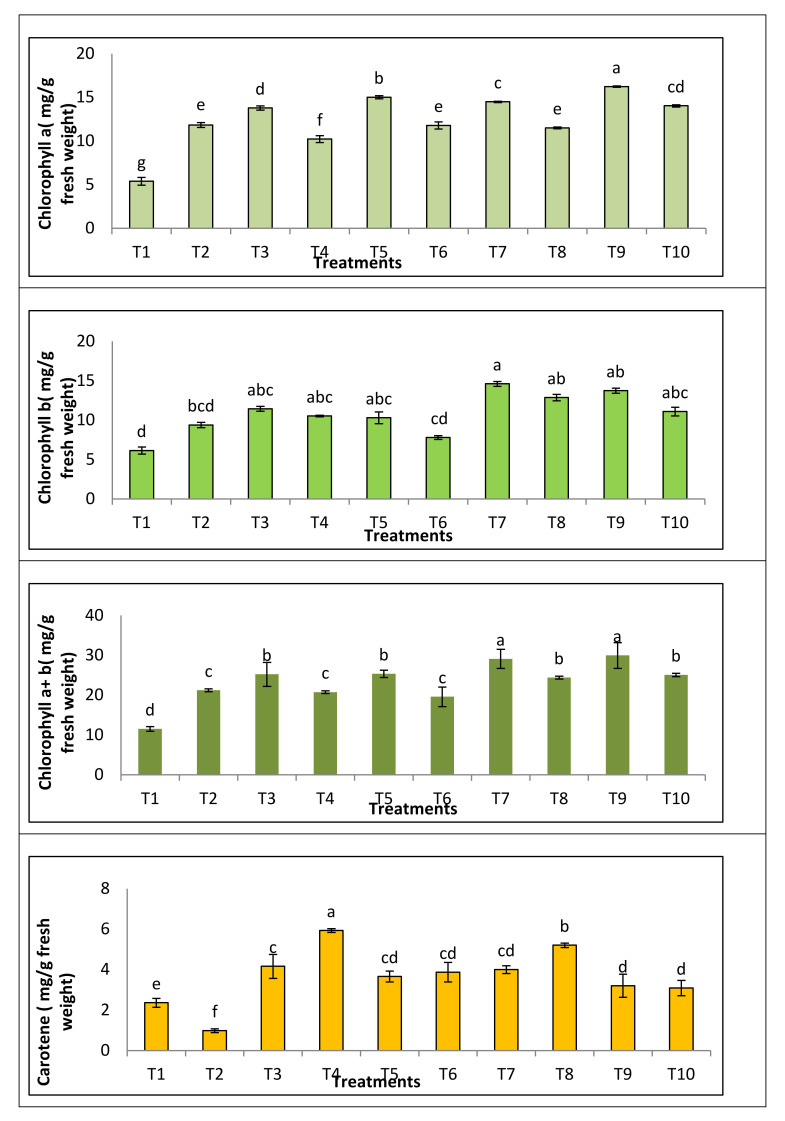
Photosynthetic pigments of tomato plant treated with PGPF. (T1) infected control, (T2) healthy control, (T3) healthy + *M. circinelloides*, (T4) infected + *M. circinelloides*, (T5) healthy + *P. oxalicum*, (T6) infected + *P. oxalicum*, (T7) healthy + *A. niger*, (T8) infected + *A. niger*, (T9) healthy + *A. flavus*, (T10) infected + *A. flavus*.

As shown in Figure 5, chlorophyll a and b contents significantly decreased, but carotenoids were raised in the *Fusarium*-infected plants. On the other hand, a significant improvement in the content of photosynthetic pigments was noticed for infected tomato plants treated with tested PGPF. Moreover, the data showed that the treatment with *M. circinelloides* strain exhibited the most potent effect in terms of the chlorophyll a content, followed by treatment with (*P. oxalicum*, *A.* *flavus* and *A. niger*). While *M. circinelloides* strain exhibited the most potent effect in chlorophyll b contents recovery, followed by treatment with *A. flavus*, *A. niger* and *P. oxalicum*, respectively. In *Fusarium* infected plant and treated with *A. niger*, *A.* *flavus*, *M. circinelloides* and *P. oxalicum*, the contents of carotenoids were markedly enhanced compared to the control. 

#### 3.3.4. Physiological and Metabolic Changes

**Figure 6:**
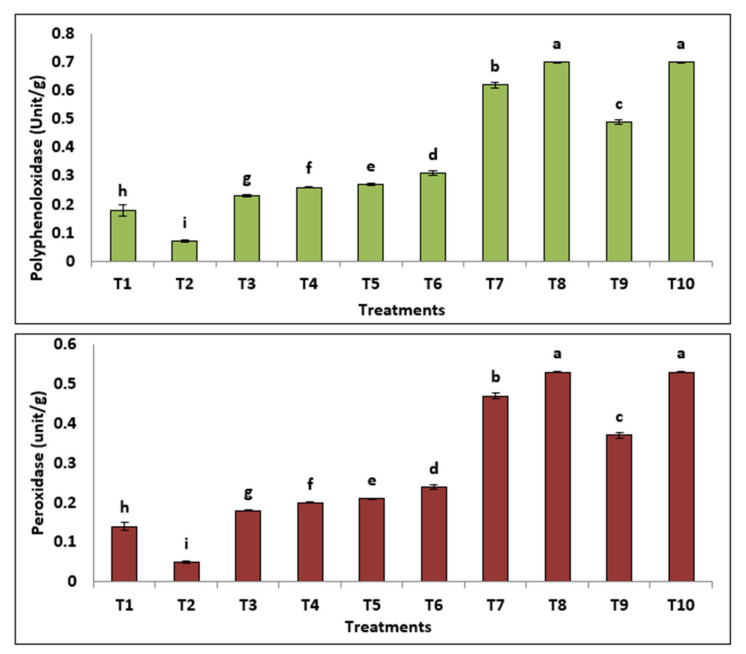
Oxidative enzymes indicators of tomato plant treated with PGPF. (T1) infected control (T2), healthy control, (T3) healthy + *M. circinelloides*, (T4) infected + *M. circinelloides*, (T5) healthy + *P. oxalicum*, (T6) infected + *P. oxalicum*, (T7) healthy + *A. niger*, (T8) infected + *A. niger*, (T9) healthy + *A. flavus*, (T10) infected + *A. flavus*.

As shown in Table 2, the results revealed that total soluble proteins and total carbohydrates of tomato declined significantly, but the total phenols and free proline of tomato increased significantly in response to *F. oxysporum* infection. It was noticed that tested elicitors (*A. niger*, *A.* *flavus*, *M. circinelloides* and *P. oxalicum*) caused enhancement of total soluble proteins and carbohydrates compared to control plants. However, pre-treatment with *M. circinelloides* resulted in the most potent significant outcome in terms of the total soluble protein and total carbohydrate. *M. circinelloides* significantly increased total phenols and free proline compared to *A. flavus*, *P. oxalicum* and *A. niger*. Moreover, Results in Figure 6 revealed that oxidative enzymes (POD and PPO) were significantly increased in infected plants. Additionally, the most significant increase in POD and PPO activity was achieved by utilizing *M. circinelloides* on the infected plants, followed by *A. flavus*, *P. oxalicum* and *A. niger*.

#### 3.3.5. Isozymes

**Figure 7:**
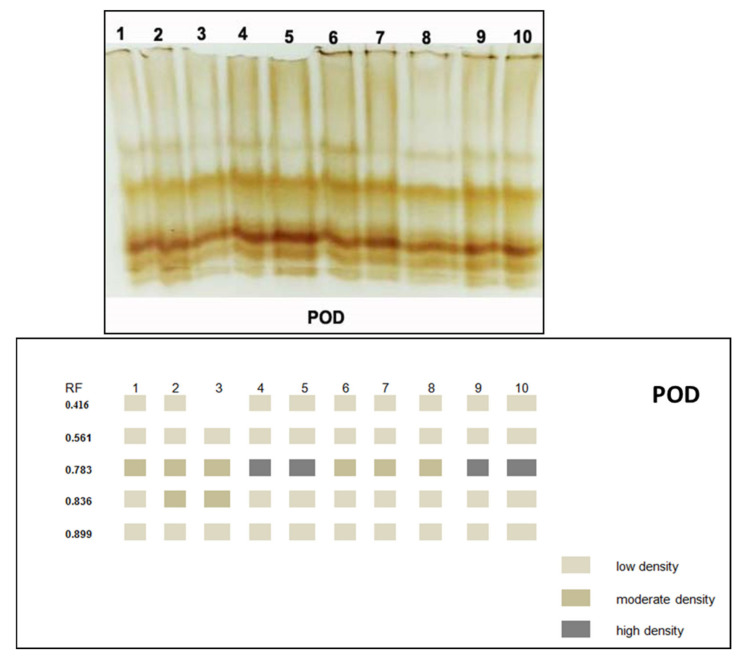
Effect of *F. oxysporum* and application of tested elicitors (*A. niger*, *A. flavus*, *P. oxalicum* and *M. circinelloides*) on POD isozyme of tomato plants.

**Figure 8:**
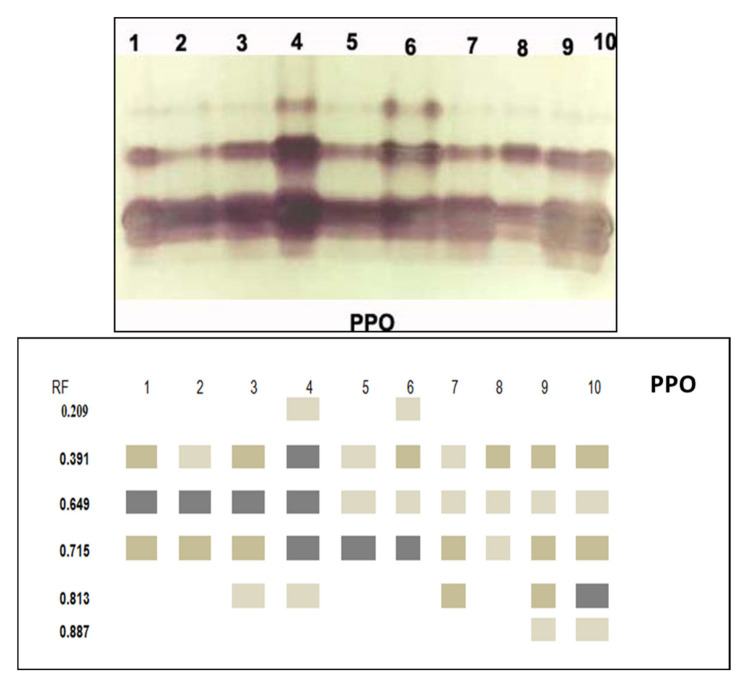
Effect of *F. oxysporum* and application of tested elicitors (*A. niger*, *A.* *flavus*, *P. oxalicum* and *M. circinelloides*) on PPO isozyme of tomato plants.

Native PAGE in Figure 7 and Figure 8 and Table 3 showed 5 POD isozymes at Rf (0.484, 0.561, 0.783, 0.836 and 0.899). Infected plants showed extremely overexpressed POD that noted5 bands containing three pale bands at Rf (0.416, 0.561 and 0.899), one reasonable band at Rf (0.836) and one extremely condensed band at Rf (0.783). Application of tested elicitors (*A. niger*, *A.* *flavus*, *P. oxalicum* and *M. circinelloides*) on infected plants recorded the same five bands at the same Rf, in which three of them were pale bands at Rf (0.484, 0.607 and 0.934), while the other two bands were modest t at Rf (0.783 and 0.836). Healthy plants treated with (*A. niger*, *A.* *flavus*, *P. oxalicum* and *M. circinelloides*) expressed the lowest POD expression that they produced two faint bands at Rf (0.416 and 0.607) and one moderate band at (0.806). On the other hand, the PPO isozyme of plant leaves showed six PPO isozymes at Rf (0.209, 0.391, 0.649, 0.715, 0.183 and 0.887). Infected plants showed highly PPO produced six bands, including two moderate bands at Rf (0.204 and 0.786) and one highly dense band at Rf (0.629). Infected plants treated with *M. circinelloides* appeared with the over expiration isozyme PPO that showed five bands recorded two moderated bands at Rf (00.391 and 0.715), two faint bands at Rf (0.649 and 0.887) and one high-density band at (0.813).

## 4. Discussion

Fungal diseases are among the most dangerous biological stresses that cause severe damage to agricultural crops in many countries [5,45]. One of the most famous pathogens of fungal diseases, *Fusarium oxysporum*, causes a negative impact on crops, especially vegetable crops [10,11,46]. Control of Fusarium wilt was difficult due to the causal agent (*Fusarium oxysporum* is a soil-borne fungus that has been found in organic and conventional agricultural soil for many years [47]. Biostimulant fungi are considered bioactive compounds reservoirs, including antimicrobial and antioxidant [46,48]. Plant growth promoting fungi (PGPF) can enhance the plant nutrient uptake from the soil and decomposes organic matter; thus, it is considered a promising strategy for sustainable development of agriculture and maintaining productivity [49]. The use of growth-stimulating microorganisms is a common strategy for researchers to enhance and improve the defense capacity and physiological immunity of plants as well as the bioavailability of minerals in the soil [15,16]. Four fungal strains, *A. flavus*, *A. niger*, *M. circinelloides* and *P. oxalicum*, were previously isolated and identified morphologically in our previous study [31]. In our previous study, these fungal strains were used for biochemical defense against fusarial infection at 60 days. Therefore, in the current study, these fungal strains were used for biocontrol of *Fusarium* wilt of tomato (45 days post inoculation). Previous studies described *P. oxalicum* and *A. niger* as PGPF [50,51,52,53]. Murali and Amruthesh [50] reported that *P. oxalicum* as PGPF could enhance plant growth and induce resistance in pearl millet against downy mildew disease. Moreover, Yin Z. et al. [52] confirmed that *P. oxalicum* promoted the growth of eggplant in new reclamation land. Another study promoted the growth and phosphate solubilization of maize in calcareous soil using *P. oxalicum* P4 and *A. niger* P85 [51]. Li, X. et al. [53]_ENREF_39 used *A. niger* for growth promotion of native forage grass. Furthermore, *M. cicinelloides* used as a plant growth promtor. Moreover, *A. flavus* was reported as PGPF, where promoted the growth of soybean and sunflower seedlings at elevated temperature [54]. Additionally, bdel-Motaal et al. [20] used *A. flavus* for growth promotion and early blight suppression in tomato. The four extracts of *M. circinelloides*, *P. oxalicum A. flavus* and *A. niger* exhibited in vitro antifungal activity. The MIC of *A. niger* was 0.25 mg/mL, while it was 0.5 mg/mL for each extract of *M. circinelloides*, *P. oxalicum* and *A. niger*. Ismail et al. [55] recorded that *Aspergillus* has antifungal activity against *Fusarium*, thus it was applied as a new bio fungicide. Moreover, Jovičić-Petrović, et al. [56] reported that *Aspergillus* has the ability to inhibit *F. oxysporum* by 33%.

Disease severity was the first guide to govern systemic resistance in treated plants by PGPF. The present results indicated that *Fusarium oxysporum* has a highly destructive effect on tomato plants by PDI 87.5%, similar to other studies studies [27,57,58]. On the other hand, all applied PGPF reduced PDI where *A. niger*, *M. circinelloides* *A. flavus* and *P.* *oxalicum* isolates reduced the percentage of disease severity by 16.6, 20.83, 37.5 and 45.83%, respectively. In addition, PGPF revealed high protection percentage of 81.3, 76.19, 59.06, 57.14 and 47.62% against *Fusarium* wilt. According to the present data, the treatment with *A. niger* strain was the best treatment in terms of reducing the PDS and recorded the highest protection. This protective effect of PGPF against *Fusarium* wilt was reported by several studies, which reported the ability of PGPF to suppress *Fusarium* pathogen [59,60].

Many beneficial functions of growth-stimulating soil microbes were reported, through stimulating ion transport to enhance vegetative growth, production of amino cephalosporanic acid acylase enzymes, resisting pathogens and stimulating plant immunity [61]. In this study, the untreated plants in the control infected with *F. oxysporum* showed significant shortage lengths of (shoot and root) and the number of leaves. These results are in agreement with Hassan et al., El-Marzoky et al., Jaber and Alananbeh [57,62], and it can be explained by *Fusarium* causes severe disturbance in growth hormones, which leads to a clear defect in cell biology [63,64]. The adverse effects may be due to disorders in the pathways of growth hormones [12,65,66,67]. Our results proved that various growth parameters (shoot length, root length and the number of leaves) were significantly increased by using the extracts of all PGPF, as shown in Figure 4. These results are explained by the ability of *A. niger*, *M. circinelloides A. flavus* and *P. oxalicum* to produce bioactive compounds such as HCN, IAA and Siderophores, and also have the ability to solubilize phosphate from soil [31]. On the other hand, infected plants treated with tested fungal isolates (*A. niger*, *A.* *flavus*, *M. circinelloides* and *P. oxalicum*) showed hopeful recovery, and the best treatment was *P. oxalicum*. These results were explained by Hussain et al., Chand et al., Ismail et al. and Hussain et al. [68,69,70,71]; they recorded the ability of these fungal isolates to secrete secondary metabolites that stimulate plant growth and stimulate substances responsible for defense.

Pathological injuries, especially fusarial infection, cause the failure of the photosynthesis process, which is considered the most important vital process within the plant [72,73]. The ability of plants to carry out the process of photosynthesis is the most important aspect of health. The present results showed that chlorophyll a and b contents were significantly decreased, but carotenoids were raised in the *Fusarium*-infected plants. Photosynthesis is the main purpose of plants, empowering them to convert light energy into chemical energy, which is next utilized in all cell activities, and it is highly altered by pathogenic infection [72,74]. This decreases in chlorophyll pigments can be explained by Choudhury and Panda, Jahan et al. and Singh et al. [75,76,77]; they mentioned that the decrease in chlorophyll is a result of oxidative stress after injury due to the release of free radicals, causing damage or distortion in the formation of chloroplasts, and this means the failure or inability of the plant to capture light and carry out the process of photosynthesis. Moreover, data showed that the treatment with *M. circinelloides* strain exhibited the most potent effect in terms of the chlorophyll a contents, followed by treatment with *P. oxalicum*, *A.* *flavus* and *A. niger.* While *M. circinelloides* strain exhibited the most potent effect in chlorophyll b contents recovery, followed by treatment with *A. flavus*, *A. niger* and *P. oxalicum*, respectively, in infected plants. In *Fusarium* infected plant and treated with *A. niger*, *A.* *flavus*, *M. circinelloides* and *P. oxalicum*, the contents of carotenoids were markedly enhanced compared to control. Our results in harmony with Jovičić-Petrović et al., Anwer and Khan, Neagu et al., Dufossé, and Al-Ani and Adhab [78,79,80,81,82], who found that *A. niger*, *P. oxalicum*, *A. flavus* and *M. circinelloides* resulted in improvement of photosynthetic pigment in plants. The positive effect in photosynthetic pigments due to treatment with inoculation (*A. niger*, *A.* *flavus*, *M. circinelloides* and *P. oxalicum*) could be attributed to enriching the plant and its soil with N_2_ element [83]. The indirect effects of PGPF(*A. niger*, *A.* *flavus*, *M. circinelloides* and *P. oxalicum*) strains in the disease suppression include the activation of the plant defense mechanisms through enhancement of photosynthetic pigments when tested with pathogens [84].

Our results revealed that total soluble proteins and total carbohydrate of tomato declined significantly, but the total phenols and free proline of tomato were increased significantly in response to *F. oxysporum* infection, which was also reported by studies from Jovičić-Petrovi et al. and Abdelaziz et al. [79,85]. The application of *A. niger*, *A.* *flavus*, *M. circinelloides* and *P. oxalicum* caused enhancement of total soluble proteins and carbohydrates compared to control plants. Total protein was determined as a response to the induction of systemic resistance [86]. The indirect effects of PGPF in disease destruction include the activation of the plant defense mechanisms through the production of proteins [87]. In line with the conclusions of phytochemistry and cell biology, we can say that physiological immunity results from many biological reactions, including changes in the cell wall and the synthesis of substances responsible for the defense, such as phytoalexins and proteins related to pathogenesis [88,89,90]. However, pre-treatment with *M. circinelloides* resulted in the most potent significant outcome in terms of the total soluble protein and total carbohydrates. Increasing carbohydrates by PGPF plays an important role in physiological protection against several pathogens through stimulate hormones and various defense pathways that caused gene expression changes [91]. The pathogen’s provocation of the plant when the invasion occurs leads to the plant producing new substances that were not present in the healthy plant or to an increase in the concentration of substances that were present before the infection. These substances work to stop the pathogen’s progress or limit its progress. Those produced may be inhibiting the growth of pathogens such as phenols and phenol oxidation products to more toxic substances [92]. It is noticeable that the greatest value recorded for the total phenols and free proline was achieved by applying *M. circinelloides* to the infected plants, followed by *A. flavus*, *P. oxalicum* and *A. niger.* Increasing total phenols plays a vital role in metabolism regulation, vegetative growth and lignin synthesis [93,94]. These results are in good agreement with those reported by Chávez-Arias et al. [95], who reported that infected tomato plants by Fusarium wilt show a distinct increase in the levels of proline. Moreover, the present data revealed that oxidative enzymes (POD and PPO) were significantly increased in infected plants [96]. Additionally, the most significant increase in POD and PPO activity was achieved by utilizing *M. circinelloides* on the infected plants, followed by *A. flavus*, *P. oxalicum* and *A. niger*. This is in concordance with Hassan et al. [97], who reported that application of PGPF caused induction of POD and PPO of Fusarium infected plants. These enzymes produce compounds that exhibit the start steps in the induction of resistance and phenolic molecules [98]. The results of this study confirm the increase in phenols, proline and antioxidant enzymes as a result of the use of plant growth-promoting fungi and are strong evidence for the induction of systemic resistance in plants. The accumulation of osmolytes and ant stress compounds serve as a common phenomenon that plays an important role in reactive oxygen species (ROS) scavenging [99], supplying plant cells with energy, as well as modulating cell redox homeostasis [100]. Isozymes are considered crucial control agents for cell metabolism in plants, and changes in isozyme profiles play a vital role in cellular defense against fungal infection [85,101]. Our results indicated that infected plants showed extremely overexpressed POD and PPO isozyme. On the other hand, the application of tested elicitors (*A. niger*, *A.* *flavus*, *P. oxalicum* and *M. circinelloides*) on infected plants recorded highly expressed POD an PPO isozymes. New isozyme bands were induced by *Fusarium* infection; thus, the antioxidant enzyme activities in *Fusarium*-infected plants treated with PGPF isolates (*A. niger*, *A.* *flavus*, *P. oxalicum* and *M. circinelloides*) were better than control plants. These results explained the major role of PGPF isolates in protecting tomato plants against *Fusarium* wilt. This enhancement of PPO and POD isozymes against disease have been recorded in the studies of Abdelaziz et al. and Harish et al. [85,102]. These enzymes produce compounds that exhibit the initial steps in the induction of plant resistance [103,104].

## 5. Conclusions

In the current study, four PGPF were identified genetically as *M. circinelloides*, *A. flavus*, *A. niger and P. oxalicum*. the identified fungal strains were evaluated for tomato growth promotion as well as control of tomato Fusarium wilt disease. In vitro results revealed that all tested isolated PGPF have promising antifungal activity against *F. oxysporum*. Moreover, in vivo results confirmed that all PGPF have the ability to stimulate the growth of healthy tomato plants and enhance the efficiency of tomato plant immunity against Fusarium wilt. Finally, these results are promising in agricultural applications as safe biological agents as alternatives strategy to chemical fungicides and as biostimulators. In the future, more studies should be conducted economically for the application of these PGPF, which can be used commercially on a large scale after its development and the completion of this study under field conditions.

## Figures and Tables

**Table 1:** Effect of PGPF on disease index of infected Tomato plants.

Treatment	Disease Symptoms Classes					DI (Disease INDEX) (%)	Protection (%)
	0	1	2	3	4		
Control Infected	0	0	0	3	3	87.5 ^a^	0
Infected + *A. niger*	3	2	1	0	0	16.6 ^e^	81.03 ^a^
Infected + *A. flavus*	2	1	1	2	0	37.5 ^c^	57.14 ^c^
Infected + *M. circinelloides*	3	1	2	0	0	20.83 ^d^	76.19 ^b^
Infected + *P. oxalicum*	2	1	1	0	2	45.83 ^b^	47.62 ^d^

**Table 2:** Metabolic indicators of tomato plant treated with PGPF.

Treatments	Total Carbohydrate	Total Phenol	Total Protein	Prolin
T1	15.03 ± 1.45 ^f^	0.74 ± 0.08 ^i^	24.05 ± 2.33 ^f^	1.33 ± 0.15 ^f^
T2	28.68 ± 0.64 ^d^	0.28 ± 0.01 ^h^	45.88 ± 1.03 ^d^	0.51 ± 0.03 ^g^
T3	23.28 ± 0.20 ^c^	0.96 ± 0.005 ^g^	51.66 ± 0.32 ^c^	1.73 ± 0.009 ^e^
T4	24.96 ± 1.07 ^e^	1.07 ± 0.01 ^f^	39.94 ± 1.71 ^e^	1.93 ± 0.01 ^d^
T5	37.38 ± 1.86 ^b^	1.14 ± 0.01 ^e^	59.97 ± 2.97 ^b^	1.76 ± 0.02 ^e^
T6	27.16 ± 0.61 ^d,e^	1.30 ± 0.03 ^d^	43.46 ± 0.98 ^d,e^	2.01 ± 0.05 ^d^
T7	28.12 ± 0.36 ^d^	2.06 ± 0.03 ^c^	44.99 ± 0.58 ^d^	3.92 ± 0.06 ^b^
T8	26.08 ± 0.68 ^d,e^	2.32 ± 0.006 ^b^	31.72 ± 1.09 ^d,e^	3.01 ± 0.008 ^a^
T9	40.12 ± 0.36 ^a^	1.63 ± 0.03 ^a^	64.19 ± 0.58 ^a^	2.12 ± 0.042 ^c^
T10	28.88 ± 2.28 ^d^	2.32 ± 0.006 ^a^	46.20 ± 3.65 ^d^	3.01 ± 0.008 ^d^
LSD at 0.05	1.98	0.057	3.17	0.099

(T1) infected control, (T2) healthy control, (T3) healthy + *M. circinelloides*, (T4) infected + *M. circinelloides*, (T5) healthy + *P. oxalicum*, (T6) infected + *P. oxalicum*, (T7) healthy + *A. niger*, (T8) infected + *A. niger*, (T9) healthy + *A. flavus*, (T10) infected + *A. flavus*.(Letters a to f revealed the significance).

**Table 3:** Isomers of POD and PPO and their retention factor (Rf) in response to *F. oxysporum*.

RF		1	2	3	4	5	6	7	8	9	10
POD	0.416	+	+	-	+	+	+	+	+	+	+
	0.561	+	+	+	+	+	+	+	+	+	+
	0.783	++	++	++	+++	+++	++	++	++	+++	+++
	0.836	+	++	++	+	+	+	+	+	+	+
	0.899	+	+	+	+	+	+	+	+	+	+
PPO	0.209	-	-	-	+	-	+	-	-	-	-
	0.391	++		++	+++	+	++	+	++	++	++
	0.649	+++	+++	+++	+++	+	+	+	+	+	+
	0.715	++	++	++	+++	+++	+++	++	+	++	++
	0.813	-	-	+	+	-	-	+	-	++	+++
	0.887	-	-	-	-	-	-	-	-	+	+

1 = Control, 2 = Control Infected, 3 = Healthy + *A. niger*, 4 = Infected *+ A. niger*, 5 = Healthy + *P. oxalicum*, 6 = Infected + *P. oxalicum*, 7 = Healthy *+ A. flavus*, 8 = Infected *+ A.* *flavus*, 9 = Healthy + *M. circinelloides*, 10 = Infected + *M. circinelloides* (- absent of band; + low density; ++ moderate density; +++ high density).

## Data Availability

Not applicable.
